# A 5′-uridine amplifies miRNA/miRNA* asymmetry in *Drosophila *by promoting RNA-induced silencing complex formation

**DOI:** 10.1186/1758-907X-2-4

**Published:** 2011-06-07

**Authors:** Hervé Seitz, Jogender S Tushir, Phillip D Zamore

**Affiliations:** 1 Laboratoire de Biologie Moléculaire Eucaryote, 118 route de Narbonne, Université Toulouse III Paul Sabatier (UPS), F-31000 Toulouse, France; 2 Laboratoire de Biologie Moléculaire Eucaryote, 118 route de Narbonne, Centre national de la recherche scientifique (CNRS), F-31000 Toulouse, France; 3 Howard Hughes Medical Institute and Department of Biochemistry and Molecular Pharmacology, University of Massachusetts Medical School, 364 Plantation street, Worcester, MA 01605, USA

## Abstract

**Background:**

MicroRNA (miRNA) are diverse in sequence and have a single known sequence bias: they tend to start with uridine (U).

**Results:**

Our analyses of fly, worm and mouse miRNA sequence data reveal that the 5′-U is recognized after miRNA production. Only one of the two strands can be assembled into Argonaute protein from a single miRNA/miRNA* molecule: in fly embryo lysate, a 5′-U promotes miRNA loading while decreasing the loading of the miRNA*.

**Conclusion:**

We suggest that recognition of the 5′-U enhances Argonaute loading by a mechanism distinct from its contribution to weakening base pairing at the 5′-end of the prospective miRNA and, as recently proposed in *Arabidopsis *and in humans, that it improves miRNA precision by excluding incorrectly processed molecules bearing other 5′-nt.

## Background

MicroRNA (miRNA) are approximately 22-nt regulatory RNA that direct members of the Argonaute protein family to their mRNA targets [1]. Together, miRNA guide and the Argonaute protein form the core of the RNA-induced silencing complex (RISC), which recognizes its mRNA targets primarily through its seed sequence, nt 2 through nt 7 [2].

The RNase III enzymes Drosha and Dicer excise most animal miRNA from long primary transcripts (pri-miRNA). Drosha cleaves pri-miRNA to release an approximately 65-nt pre-miRNA; Dicer cleaves the pre-miRNA to liberate a miRNA/miRNA* duplex. The duplex is then loaded into an Argonaute protein. The geometry of the miRNA/miRNA* duplex during the loading reaction determines the fate of each small RNA: the miRNA binds tightly to Argonaute, with its 5′-nt anchored in a positively charged pocket in the Mid domain of the protein [3,4]. The miRNA* assumes the same position as subsequent mRNA targets and is held to the complex predominantly by seed sequence base pairing. A seed sequence mismatch between the miRNA and its miRNA* is believed to promote miRNA* dissociation [5,6]. A subset of Argonaute proteins can cleave the miRNA* if it is extensively paired to the miRNA, triggering its destruction [7-10]. The orientation of the duplex during Argonaute loading is not random: the miRNA is usually the strand with the less stably paired 5′-end in the duplex [11,12]. Consequently, the duplex liberated by Dicer determines the identity of the miRNA.

miRNA sequences are diverse, and only one common sequence motif has been identified. Most miRNA begin with a 5′-uridine (5′-U). In plants, a 5′-U directs miRNA to AGO1, small RNA that begin with adenosine (A) load AGO2 and those that start with cytidine (C) load AGO5 [13-15]. Likewise, the 5′-nt of fly small RNA participates in sorting, with a 5′-U directing small RNA toward Ago1 and a 5′-C favoring Ago2 [16-19]. In mammals, the Mid domain of Ago2, the homolog of *Drosophila *Ago1, specifically recognizes a 5′-U or 5′-A [20], explaining why miRNA tend to start with those nucleotides, but fly and worm miRNA typically begin with 5′-U but not 5′-A. Moreover, small RNA sorting in flies and worms also reflects the secondary structure of the miRNA/miRNA* duplex, with centrally paired duplexes preferentially loaded into one Argonaute, - Ago2 in flies and RDE-1 in worms, - and duplexes bearing a central mismatch directed toward the major miRNA-binding Argonautes, - Ago1 in flies and the paralogous ALG-1/ALG-2 proteins in worms [5,6,17-19,21-23].

We investigated the function of 5′-U in animal miRNA. Our statistical analyses of sequencing data from flies, worms and mice reveal that 5′-U is recognized after miRNA/miRNA* production by Dicer cleavage of the pre-miRNA. Our experimental results show that 5′-U facilitates loading of miRNA while decreasing loading of miRNA*, consistent with the view that only one of the two strands can be assembled from a single miRNA/miRNA* molecule. Our data support the view that 5′-U enhances RISC assembly by a mechanism distinct from its contribution to destabilizing base pairing at the 5′-end of miRNA. Similarly to what has been proposed in *Arabidopsis thaliana *and in *Homo sapiens *[13,20], our data also suggest that recognition of the first miRNA nucleotide during loading may select against incorrectly processed molecules bearing 5′-nt other than 5′-U.

## Results and discussion

### 5′-U acts after miRNA processing

**Figure 1:**
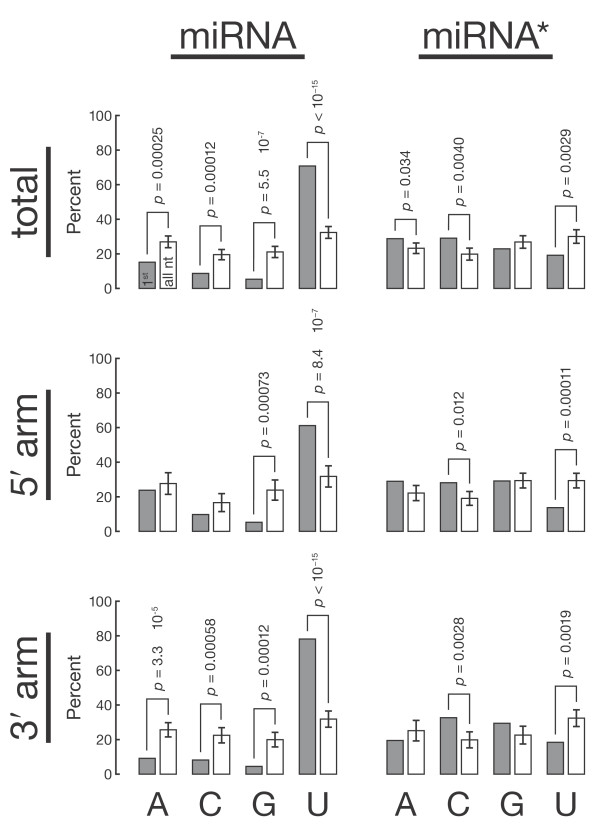
**Fly miRNA tend to start with U**. Each miRNA or miRNA* isoform derived from a common pre-miRNA was weighted according to its abundance in the pooled deep-sequencing libraries, and the sequence composition analyses for all small RNA from different pre-miRNA that were read at least 100 times in the pooled libraries were weighted equally. Gray, nucleotide frequency at position 1; white, 100 sets of nucleotides randomly selected from nt 1-18 of the miRNA and miRNA* species to assess the overall nucleotide composition of miRNA and miRNA*. Each random set had the same size as the corresponding set of miRNA or miRNA* 5′-nt. *P *values measure the probability of picking a random set from nt 1-18 with the same nucleotide frequency as the actual set of 5′-nt.

We used high-throughput sequencing data to examine the 5′-sequence bias of miRNA and miRNA*. miRNA are far more likely to begin with U in flies (*P *value <10^-15^), worms (*P *value <10^-15^) or mice (*P *value = 1.1 × 10^-14^) than would be expected from their general nucleotide composition (Figure 1, Additional file 1, Figure S1, and Additional file 2, Figure S2). Conversely, miRNA* were less likely than expected to begin with U in flies (*P *value = 0.0029), worms (*P *value = 0.017) or mice (*P *value = 0.0020).

In theory, a 5′-U might facilitate Drosha cleavage of the pri-miRNA or pre-miRNA export from the nucleus. Such a role for a 5′-U would be reflected in a greater likelihood of both miRNA and miRNA* derived from the 5′-arm of the pre-miRNA stem to begin with U compared to those residing in the 3′ arm. We compared the approximately 40% of fly, 35% of worm and 50% of mouse miRNA that reside in the 5′-arm of their pre-miRNA to their 3′ counterparts. Our analysis argues against a role for a 5′-U in Drosha processing or nuclear export. miRNA tend to start with a U, regardless of their position in the pre-miRNA (Figure 1, Additional file 1, Figure S1, and Additional file 2, Figure S2). Moreover, miRNA* sequences tend not to begin with U, even when they derive from the pre-miRNA 5′-arm. Our data similarly exclude a role for a 5′-U in cleavage of the pre-miRNA by Dicer, which would favor a 5′-U for miRNA and miRNA* derived from the 3′-arm.

### miRNA asymmetry correlates with first nucleotide identity

**Figure 2:**
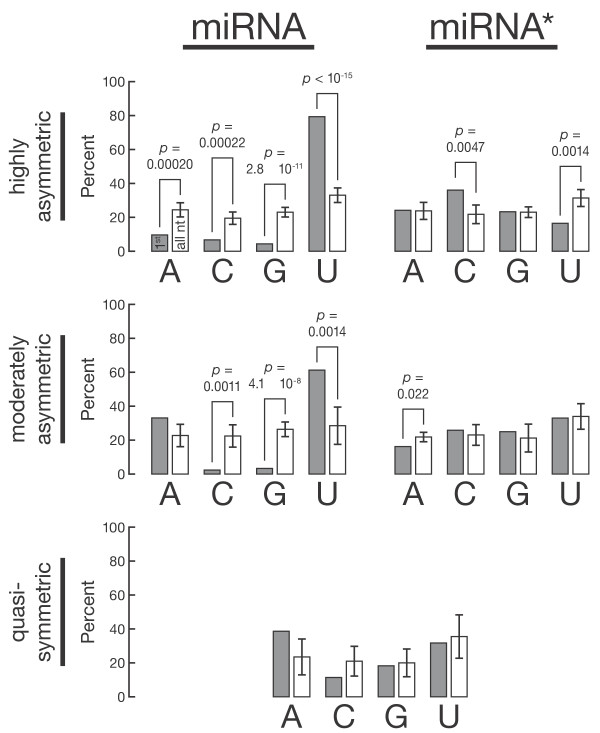
**Fly miRNA asymmetry correlates with the identity of the first nucleotide of the small RNA**. miRNA/miRNA* duplexes were binned according to their asymmetry: highly asymmetric, miRNA/miRNA* ≥10 in the pooled deep-sequencing libraries; moderately asymmetric, 10 > miRNA/miRNA* ≥ 2; quasisymmetric: miRNA/miRNA* <2; and analyzed as in Figure 1.

To test whether 5′-U plays a role in assembling a miRNA into RISC, we separately evaluated the 5′-nt frequencies in flies of highly asymmetric duplexes (miRNA/miRNA* ≥10; 79 duplexes), moderately asymmetric duplexes (2 < miRNA/miRNA* < 10; 33 duplexes) and quasisymmetric duplexes (miRNA/miRNA* < 2; 10 duplexes). If the identity of the 5′-nt affects miRNA loading, then the most asymmetric miRNA should exhibit a higher 5′-U bias than the least asymmetric miRNA. Indeed, the most highly asymmetric miRNA have a higher frequency of 5′-U (79%) than moderately asymmetric miRNA (61%) or quasisymmetric miRNA and miRNA* (32%) (Figure 2), which is in line with the previously published observation that the most asymmetric human miRNA tend to be richer in 5′-U [24]. Moreover, miRNA* strands from highly asymmetric duplexes have a significantly lower frequency of 5′-U (16.5%) than those from moderately asymmetric or quasisymmetric duplexes. In fact, miRNA* strands have a significantly lower frequency of U at their 5′-ends than across their entire sequence, while the frequency of an initial U was indistinguishable from the overall U frequency in miRNA* from moderately asymmetric or quasisymmetric duplexes.

Strikingly, the most asymmetric miRNA also exhibit a lower than expected frequency of 5′-A (Figure 2, top left), whereas the thermodynamic stability rule would have predicted a high frequency of both U and A. This observation suggests that 5′-nt identity, not just thermodynamic asymmetry, contributes to the differential loading of miRNA and miRNA* *in vivo*.

### Initial nucleotide identity influences miRNA loading *in vitro*

**Figure 3:**
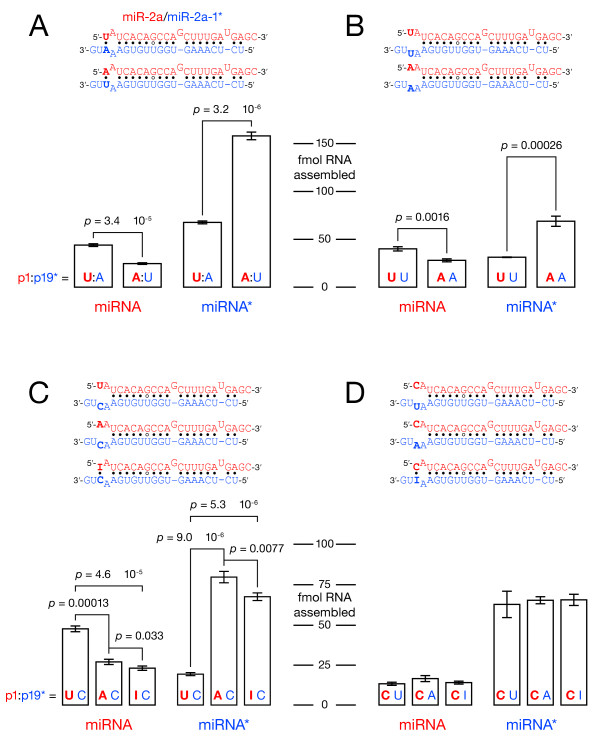
**Identity of the first miRNA nucleotide affects duplex asymmetry**. RNA-induced silencing complex (RISC) loading of miRNA and miRNA* strands was measured after *in vitro *assembly. Data are reported as means ± standard deviation for three independent replicate experiments. **(A) **Swapping the terminal uridine:adenosine (U:A) pair of the miR-2a/miR-2a-1* duplex decreased miRNA loading and increased miRNA* loading. **(B) **The effect of the terminal A:U pair mainly reflects the identity of the first miRNA nucleotide, which affects both miRNA and miRNA* loading **(C)**, whereas the identity of the facing miRNA* nucleotide has no detectable effect **(D)**.

Several studies have proposed that a U at the 5′-end of a small RNA directly promotes its loading into Ago1 in flies [18,19,25,26]. We measured the effect of initial nucleotide identity on the efficiency of loading of the miR-2a/miR-2a-1* duplex in *Drosophila *embryo lysate. To avoid altering the thermodynamic stability of the 5′-ends of the duplex, we designed them so that changing the 5′-nt preserved the pattern and strength of base pairing. To measure the association of miR-2a and miR-2a-1* with mature RISC, we assembled RISC in *Drosophila *embryo lysate using a duplex in which one strand was 5′-^32^P-radiolabeled, then captured the radiolabeled strand using a complementary 2′-*O-*methyl oligonucleotide tethered to a magnetic bead (Figure 3). Labeling either the miRNA or the miRNA* strand (always capturing RISC with an oligonucleotide complementary to the labeled strand), we were able to quantify precisely both miRNA and miRNA* loading by scintillation counting. Ultraviolet cross-linking and RISC capture control experiments demonstrated that the amount of radioactivity captured minus the amount recovered when the duplex was incubated with *N*-ethylmaleimide (NEM)-inactivated lysate reflected the amount of single-stranded miRNA or miRNA* produced by assembly of Ago1 RISC (Additional file 3, Figure S3, and Additional file 4, Figure S4).

Both authentic miR-2a and miR-2a-1* begin with U; the 5′-U of miR-2a is paired to A19 of miR-2a-1*. Inverting this U:A base pair so that miR-2a began with A nearly halved the amount of miRNA assembled into RISC and more than doubled the amount of miR-2a-1* (Figure 3A). Thus, a change in the identity of the first nucleotide of the miRNA decreased the efficiency of assembly of the miRNA into RISC and increased assembly of the miRNA* while preserving the relative thermodynamic asymmetry of the duplex.

When the initial U:A base pair of miR-2a/miR-2a-1* was altered, UU assembled more miRNA into RISC than did AA (Figure 3B). Notably, an AA mismatch at the 5′-end of the miRNA more than doubled the amount of miRNA* incorporated into RISC. Next, we examined a series of miR-2a/miR-2a* derivatives in which the 19th base of miR-2a* was always C, ensuring that duplex stability was the same when the miRNA began with U or A. Again, a 5′-U favored miRNA loading and disfavored miRNA* loading (Figure 3C). When the 5′-U was replaced with inosine, which can pair to the miRNA* C at position 19, only slightly less miRNA was assembled into RISC than that observed for an A/C mismatch. We conclude that the identity of the first miRNA nucleotide contributes more to the loading of miR-2a than do differences in the stability of the duplex termini. Reciprocally, when the first miRNA nucleotide was C, the identity of miRNA* nt 19 did not have any significant effect on miRNA or miRNA* loading (Figure 3D), demonstrating that the effect shown in Figure 3A reflects a mutation of the first miRNA nucleotide, not the change in miRNA* nt 19. Experiments using miR-14 and miR-184 gave similar results (Additional file 5, Figure S5).

Strikingly, the order of preference for nt 1 was not the same across the three tested miRNA: miR-2a preferred U > A > C (Figure 3), miR-14 preferred U ~ C > A and miR-184 preferred U ~ A > C (Additional file 6, Figure S6). Hence additional features in the miRNA/miRNA* duplex must influence the order of preference for miRNA nt 1. Mutating the overhanging nucleotide in miR-184* did not alter the efficiency of loading miR-184 (Additional file 7, Figure S7), excluding a role for base pairing between nt 1 and the 3′ overhang of the miRNA*.

### Covarying features in miRNA/miRNA* duplexes suggest that the identity of nt 2 affects the order of preference for miRNA nt 1

**Figure 4:**
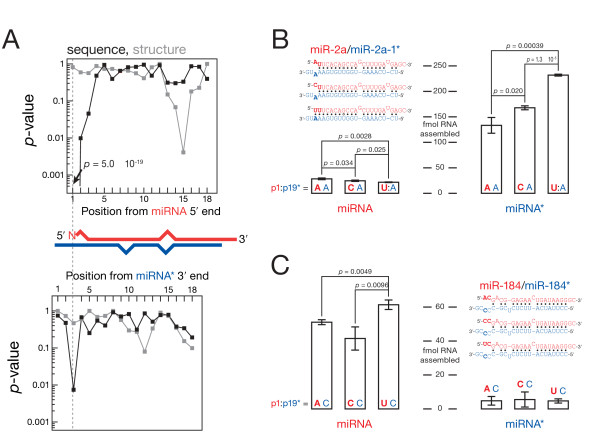
**In *Drosophila*, the identity of the second miRNA nucleotide influences the effect of the first nucleotide**. **(A) **The *y*-axis shows the significance (Fisher's exact test) of observed covariation between the identity of miRNA nt 1 and nucleotide identity (black) or base-pairing status (gray) along the miRNA and the miRNA* strand. The low values for position 1 in the black curve occur because the identity of miRNA nt 1 correlates strongly with itself. **(B and C) **Mutating nt 2 in miR-2a **(B) **or miR-184 **(C) **changed the order of nt 1 preference for miRNA loading.

If a sequence or structural feature affects the order of preference for nt 1, then these two features should evolve together. We searched for significant covariation between nt 1 identity and other sequence or structural motifs in miRNA/miRNA* duplexes. For *Drosophila *miRNA/miRNA*, the identity of miRNA nt 1 covaries with the identity of the facing nucleotide on the miRNA* strand, the identity of the second nucleotide of the miRNA strand and the base-pairing status of the 15th nucleotide of the miRNA strand (Figure 4A). Mutating miRNA nt 2 in miR-2a and miR-184 influenced the order of preference for nt 1 in flies (Figures 4B and 4C).

Strikingly, the influence of nt 2 on nt 1 seems to be specific for flies. Neither worm nor mouse miRNA/miRNA* show such covariation (Additional file 8, Figure S8). *Caenorhabditis elegans *miRNA nt 1 covaries mostly with the base-pairing status of miRNA nt 18 and the identity of the miRNA* nucleotide facing miRNA nt 3. In mouse, nt 1 covaries with the identity of miRNA nt 12 as well as several positions at the 3′ end of the miRNA strand. The sequence composition of miRNA differs greatly between flies and humans [24], suggesting that the nucleotide preference of the miRNA loading machinery has evolved since the divergence of protostomes and deuterostomes, with only the overall tendency for miRNA to start with U remaining conserved.

## Conclusions

Our data support the view that a U at the 5′-end of a miRNA favors RISC loading in flies and, given both our informatics data and the broad phylogenetic conservation of the 5′-U bias among miRNA in worms and mice, likely in animals generally.

The *Drosophila *Ago1 loading machinery remains to be identified, although chaperones have been implicated in assembling miRNA into RISC [6,27,28]. It is tempting to speculate that the requirement for the miRNA 5′-end to be the less thermodynamically stable in a miRNA/miRNA* duplex reflects the need for the first nucleotide to be single-stranded to present it to components of the RISC loading machinery or to Ago1 itself.

Why has the miRNA pathway evolved to prefer a 5′-U? The likely answer is that preferential loading of miRNA starting with U improves the precision of the miRNA 5′-end [13]. Drosha and Dicer generate pools of miRNA/miRNA* duplexes with alternative 5′- and 3′-ends; loading of these duplexes into *Drosophila *Ago2, -which prefers 5′-C, - has been shown to purify this population of miRNA [29], loading preferentially the miRNA isoforms bearing a 5′-C [19,25]. The preference of the Ago1 loading machinery or of Ago1 itself for 5′-U could similarly restrict entry into the Ago1 pathway by loading only miRNA isoforms that begin with U. Consistent with this idea, the pre-miRNA nucleotides flanking miRNA nt 1 tend to be depleted in U (Additional file 9, Figure S9). Such a purifying selection could ensure that most mature miRNA have the correct 5′-end and therefore the correct seed sequence, ensuring that they regulate the appropriate mRNA targets.

## Methods

*In vitro *reconstitution of miRNA/miRNA* loading 5′ phosphorylated miRNA/miRNA* (approximately 20 nM; the strand measured was ^32^P-radiolabeled) was incubated with zero- to two-hour fly embryo lysate for one hour at 25°C [30]. Assembly was stopped with NEM [7]. Two-thirds of each assembly reaction were incubated with biotinylated 2′-*O*-methyl capture oligonucleotide (Table 1) tethered to streptavidin-coated magnetic beads (MyOne Streptavidin C1 DYNAL Magnetic Beads; Invitrogen Corp., Carlsbad, CA, USA) for one hour at 25°C. The radioactivity in the remaining one-third of each reaction was measured by scintillation counting to allow data normalization. Typical replicate-to-replicate variability (standard deviation/mean) was approximately 5%. *P *values were calculated using Student's *t*-test assuming equal variances, and distribution normality and homogeneity of variances were assessed using the Shapiro-Wilk test and Levene's test.

**Table 1:** Synthetic oligonucleotides used in this study^a^

Oligonucleotide	Sequence (5′ to 3′)
miR-2a with P1 U	UAU CAC AGC CAG CUU UGA UGA GC
miR-2a with P1 A	AAU CAC AGC CAG CUU UGA UGA GC
miR-2a with P1 I	IAU CAC AGC CAG CUU UGA UGA GC
miR-2a with P1 C	CAU CAC AGC CAG CUU UGA UGA GC
miR-2a with P1 G	GAU CAC AGC CAG CUU UGA UGA GC
miR-2a-1* with P19 A	UCU CAA AGU GGU UGU GAA AUG
miR-2a-1* with P19 U	UCU CAA AGU GGU UGU GAA UUG
miR-2a-1* with P19 C	UCU CAA AGU GGU UGU GAA CUG
miR-2a-1* with P19 I	UCU CAA AGU GGU UGU GAA IUG
miR-184 with P1 U	UGG ACG GAG AAC UGA UAA GGG C
miR-184 with P1 A	AGG ACG GAG AAC UGA UAA GGG C
miR-184 with P1 C	CGG ACG GAG AAC UGA UAA GGG C
miR-184 with P1 T	TGG ACG GAG AAC UGA UAA GGG C
miR-184 with P1 G	GGG ACG GAG AAC UGA UAA GGG C
miR-184* with P19	CCU UAU CAU UCU CUC GCC CCG
miR-184* with P19	CCU UAU CAU UCU CUC GCC ACG
miR-184* with P21	CCU UAU CAU UCU CUC GCC CCC
miR-184* with P21 U	CCU UAU CAU UCU CUC GCC CCU
miR-184* with P21 A	CCU UAU CAU UCU CUC GCC CCA
miR-14 with P1 U	UCA GUC UUU UUC UCU CUC CUA
miR-14* with P1 A	GGA GCG AGA CGG GGA CUC ACU
miR-14 with P1 A	ACA GUC UUU UUC UCU CUC CUA
miR-14* with P19 U	GGA GCG AGA CGG GGA CUC UCU
miR-2c with P1 U	UAU CAC AGC CAG CUU UGA UGG GC
miR-2c* with P20 A	CAU CAA AAA GGG CUG AAG AAA G
Oligo to capture miR-2a and miR-2c	Bio-mAmUmGmU mUmGmG mCmUmC mAmUmC mAmAmA mGmCmU mGmGmC mUmGmU mGmAmU mCmUmG mCmUmG mA
Oligo to capture miR-2a-1*	Bio-mAmUmG mUmUmG mCmAmC mUmUmC mAmCmA mAmCmC mAmCmU mUmUmG mAmGmA mUmGmC mUmGmA
Oligo to capture miR-184	Bio-mAmUmG mUmUmG mGmCmC mCmUmU mAmUmC mAmGmU mUmCmU mCmCmG mUmCmC mCmUmG mCmUmG mA
Oligo to capture miR-184*	Bio-mAmUmG mUmUmG mCmGmG mGmGmC mGmAmG mAmGmA mAmUmG mAmUmA mAmGmG mUmGmC mUmGmA
Oligo to capture miR-14	Bio-mAmUmG mUmUmG mUmAmG mGmAmG mAmGmA mGmAmA mAmAmA mGmAmC mUmGmC mUmGmC mUmGmA
Oligo to capture miR-14*	Bio-mAmUmG mUmUmG mAmGmC mGmAmG mUmCmC mCmCmG mUmCmU mCmGmC mUmCmC mUmGmC mUmGmA
Oligo to capture miR-2c*	Bio-mAmUmG mUmUmG mCmUmU mUmCmU mUmCmA mGmCmC mCmUmU mUmUmU mGmAmU mGmUmG mCmUmG mA
pre-miR-2a-1 loop (extended by 4 nt)	CAU UUC CGC UUU GCG CGG CAU AUC
miR-2a (shortened by 4 nt)	ACA GCC AGC UUU GAU GAG C
DNA splint for pre-miR-2a-1 ligation	GCT AAG CTC ATC AAA GCT GGC TGT GAT ATG CCG CGC AAA GCG GAA ATG CAT TTC ACA ACC ACT TTG AGA GCT TA
pre-miR-2a-1	UCU CAA AGU GGU UGU GAA AUG CAU UUC CGC UUU GCG CGG CAU AUC ACA GCC AGC UUU GAU GAG C
miR-2a with U at position 1 and U at position 2	UUU CAC AGC CAG CU UUG AUG AGC
miR-2a with A at position 1 and U at position 2	AUU CAC AGC CAG CUU UGA UGA GC
miR-2a with C at position 1 and U at position 2	CUU CAC AGC CAG CUU UGA UGA GC
miR-184 with U at position 1 and C at position 2	UCG ACG GAG AAC UGA UAA GGG C
miR-184 with A at position 1 and C at position 2	ACG ACG GAG AAC UGA UAA GGG C
miR-184 with C at position 1 and C at position 2	CCG ACG GAG AAC UGA UAA GGG C

^a^I, inosine; Bio, biotin; mN, 2′-*O*-methyl ribose.

### Covariation analysis

miRNA with ≥100 reads in the pooled deep-sequencing libraries were selected (see Table 2 for the list of analyzed deep-sequencing libraries). The most abundant isoform of each strand was retained. We evaluated the identity and base-pairing status (using RNAcofold, part of the Vienna RNA Secondary Structure Package; available at http://www.tbi.univie.ac.at/RNA/) of each of the first 18 nt. If the pairing probability of a nucleotide was >0.5, it was called paired. The analysis defined 18 nt identities, starting from either the 5′- or the 3′-end, and 18 base-pairing statuses, starting from either the 5′- or the 3′-end, with a total of 144 features per miRNA/miRNA* duplex. Fisher's exact test was used to evaluate the significance of covariation between these 144 features and the identity of the first miRNA nucleotide using the R Project for Statistical Computing statistical package (http://www.r-project.org/).

**Table 2:** High throughput sequencing data used in this study^a^

Species	Accession Number
*Caenorhabditis elegans*	GSM139137, GSM297742, GSM297743, GSM297744, GSM297745, GSM297746, GSM297747, GSM297748, GSM297750, GSM297751
*Drosophila melanogaster*	GSM180328, GSM180329, GSM180330, GSM180331, GSM180332, GSM180333, GSM180334, GSM180335, GSM180336, GSM180337, GSM239041, GSM239052, GSM239054, GSM239056, GSM240749, GSM246084, GSM272651, GSM272652, GSM272653, GSM275691, GSM280082, GSM280085, GSM286602, GSM286603, GSM286604, GSM286605, GSM286606, GSM286607, GSM286611, GSM286613, GSM322208, GSM322219, GSM322245, GSM322338, GSM322533, GSM322543, GSM343832, GSM343833, GSM360256, GSM360257, GSM360260, GSM360262, GSM361908, GSM364902, GSM371638, GSM385744, GSM385748, GSM385821, GSM385822, GSM399100, GSM399101, GSM399105, GSM399106, GSM399107, GSM399110, GSM609217, GSM609218, GSM609219, GSM609220, GSM609221, GSM609222, GSM609223, GSM609224, GSM609225, GSM609226, GSM609227, GSM609228, GSM609229, GSM609234, GSM609235, GSM609238, GSM609239, GSM609240, GSM609241, GSM609242, GSM609243, GSM609244, GSM609246, GSM609247, GSM609248, GSM609249, GSM609250, GSM609251
*Mus musculus*	GSM237107, GSM237110, GSM261957, GSM261959, GSM304914, GSM314552, GSM314558

^a^Datasets were obtained from the GEO database (http://www.ncbi.nlm.nih.gov/geo/). Reads (18 to 30 nt) matching known miRNA or miRNA sequences were identified, tolerating up to 9 nt between the observed extremities and the 5′ and 3′ extremities described in miRBase version 14. Overall, the fly libraries contained 100,603,194 miRNA and 6,569,021 miRNA* reads, the worm libraries contained 14,479,717 miRNA and 1,124,773 miRNA* reads and the mouse libraries contained 3,416,073 miRNA and 143,617 miRNA* reads.

## Competing interests

PDZ is a cofounder and member of the scientific advisory board of Alnylam Pharmaceuticals, Inc., and a member of the scientific advisory board of Regulus Therapeutics, L.L.C.

## Authors' contributions

HS and PDZ planned the experiments and wrote the manuscript. HS and JST performed the experiments. All authors read and approved the final manuscript.

## Supplementary Material

Additional file 1 **Figure S1. *Caenorhabditis elegans *miRNA tend to start with a uridine**. Gray, nucleotide frequency at position 1; white, nucleotide frequency at random positions in the miRNA or miRNA* sequence (means ± standard deviation (SD)). Click here for file

Additional file 2 **Figure S2. Mouse miRNA tend to start with a uridine**. Gray, nucleotide frequency at position 1; white, nucleotide frequency at random positions in the miRNA or miRNA* sequence (means ± SD). Click here for file

Additional file 3 **Figure S3. Faithful *in vitro *reconstitution of miRNA loading. (A and B) **miR-2a, *let-7*, miR-2c and miR-184 are correctly loaded into Ago1 in fly embryo lysate, and a *let-*7/anti-*let-7 *small interfering RNA is correctly loaded into Ago2. Left: lysate prepared from embryos from *dcr-2^L811fsX^*- and *ago2^414^*-mutant mothers; Ago1-depleted and HA-depleted, wild-type embryo lysate-immunodepleted using anti-Ago1 or anti-HA (hemagglutinin epitope) antibody. Lysate was incubated for one hour with 5′-^32^P-radiolabeled *Drosophila melanogaster *miR-2a paired with 5′ phosphorylated miR-2a-1*. Single-stranded (ssRNA), 5′-^32^P-radiolabeled miR-2a was incubated for one hour in embryo lysate. Each sample was cross-linked using 254 nm ultraviolet light. **(C and D) **miRNA/miRNA* asymmetry is recapitulated *in vitro*. **(E) **In S2 cells, pre-miR-2a-1 liberates both miRNA and miRNA*, and both strands are efficiently loaded into RNA-induced silencing complex as we observed *in vitro *(Figure 3). Click here for file

Additional file 4 Figure S4. The Ago2 loading machinery has a moderate effect on miR-184 loading preferences, while it strongly affects miR-184* loading preferences. Left: miR-184 and miR-184* capture assay in *dcr-2^L811fsX^*-null mutant embryo lysate. Right: miR-184 and miR-184* capture assay in wild-type lysate. Click here for file

Additional file 5 **Figure S5. Identity of miRNA nucleotide 1 affects duplex asymmetry**. **(A) **Changing the 5′-uridine (5′-U) of miR-14 to 5′-adenosine (5′-A) decreased miRNA loading. **(B) **Changing the 5′-U of miR-184 into 5′-cytidine (5′-C) decreased miRNA loading; mutating it to ribothymidine increased miRNA loading. Changing the 5′-nt of miR-2a **(C) **or miR-184 **(D) **into 5′-guanidine (5′-G) decreases miRNA loading (relatively to a 5′-A). Click here for file

Additional file 6 **Figure S6. Duplex-specific order of preference on the identity of the first nucleotide**. **(A) **Regardless of the identity of the facing (p19*) nucleotide, miR-2a is better loaded if it starts with a U than if it starts with an A than if it starts with a C (U > A > C). **(B) **miR-14 is better loaded if it starts with a U or a C than if it starts with an A (U ~ C > A). **(C) **miR-184 is better loaded if it starts with a ribothymidine than if it starts with a U or an A than if it starts with a C (T > U ~ A > C). Click here for file

Additional file 7 Figure S7. The sequence of the miRNA* 3′ overhang is not responsible for miRNA-specific preferences for nt 1. The modest effect of the identity of the miRNA* 3′-most nucleotide does not correlate with the base-pairing ability of miRNA nt 1 to the miRNA* 3′ terminus. Click here for file

Additional file 8 **Figure S8. Structure and sequence features covarying with the identity of miRNA nt 1 in *C. elegans *and mouse**. See Figure 4A legend for details. **(A) **Covariation in *Mus musculus *miRNA/miRNA* duplexes. **(B) **Covariation in *C. elegans *miRNA/miRNA* duplexes. Click here for file

Additional file 9 **Figure S9. Pre-miRNA nucleotides flanking miRNA nt 1 are depleted of U**. U frequency was measured in pre-miRNA covered by at least 100 reads in the pooled deep-sequencing libraries. The 5′-most nucleotide of mature miRNA is enriched in U (position 0 on the *x*-axis), while its flanking nucleotides are depleted. The horizontal line indicates the mean U frequency in 100 random sets of nucleotides picked from the corresponding 21-nt segment in the analyzed pre-miRNA. Dashed lines indicate 95% confidence intervals. Click here for file
