# Supplementary figures and images for: A 5′-uridine amplifies miRNA/miRNA* asymmetry in Drosophila by promoting RNA-induced silencing complex formation
Source: Silence. 2011 Jun 7;2:4. doi: 10.1186/1758-907X-2-4 (PMC3127740; doi:10.1186/1758-907X-2-4)

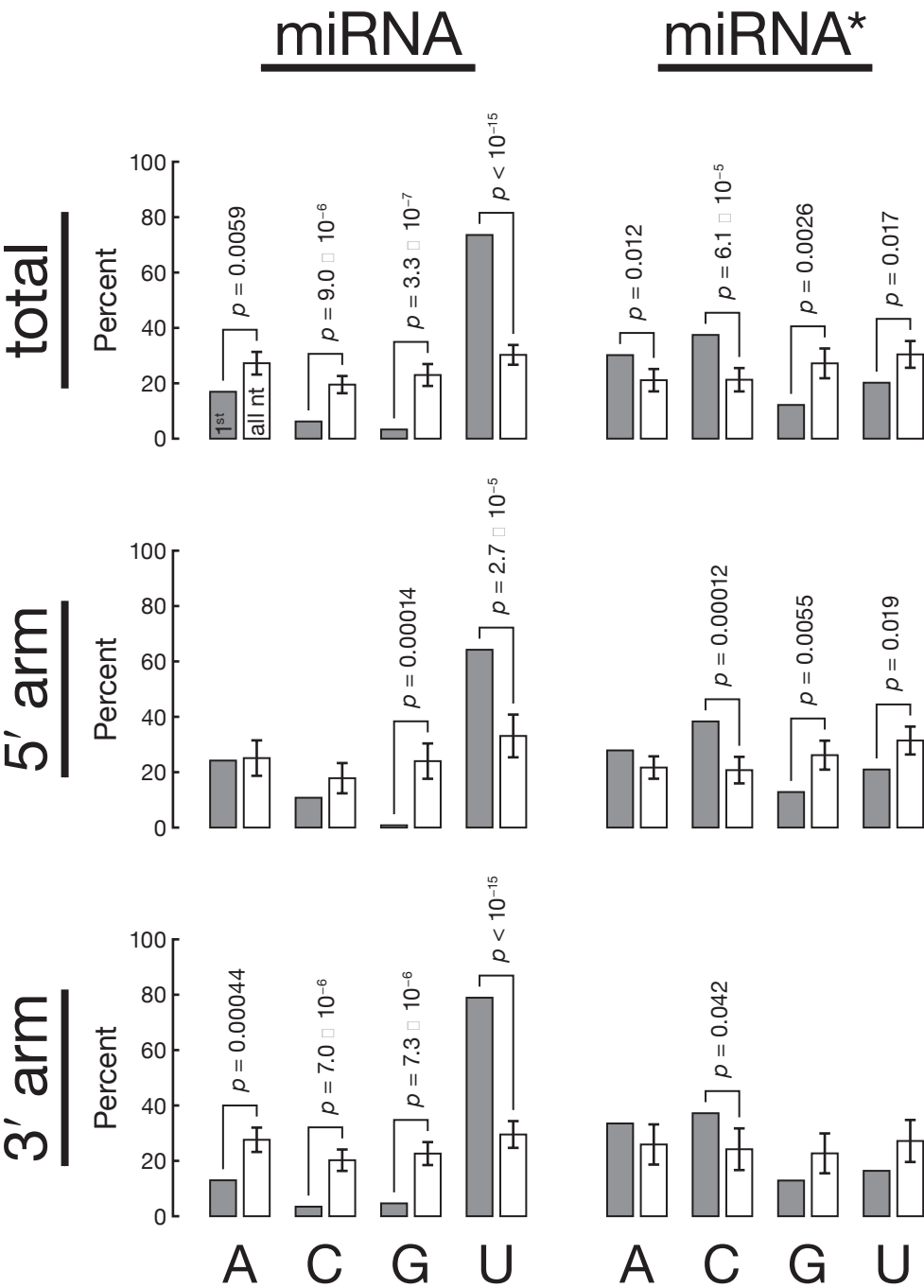

Supplement: Additional file 1 — Figure S1. Caenorhabditis elegans miRNA tend to start with a uridine. Gray, nucleotide frequency at position 1; white, nucleotide frequency at random positions in the miRNA or miRNA* sequence (means ± standard deviation (SD)). [file 1758-907X-2-4-S1.PDF]

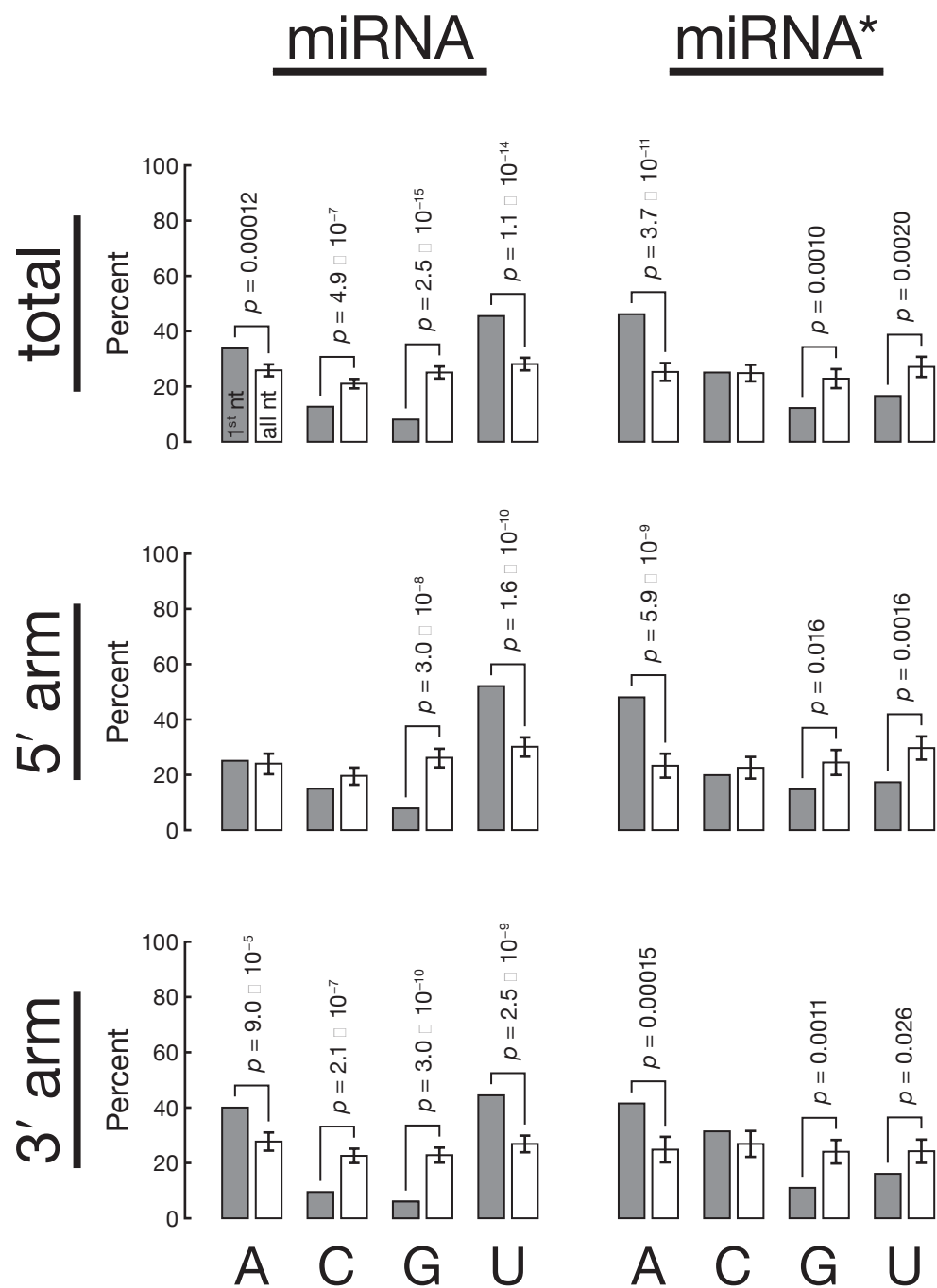

Supplement: Additional file 2 — Figure S2. Mouse miRNA tend to start with a uridine. Gray, nucleotide frequency at position 1; white, nucleotide frequency at random positions in the miRNA or miRNA* sequence (means ± SD). [file 1758-907X-2-4-S2.PDF]

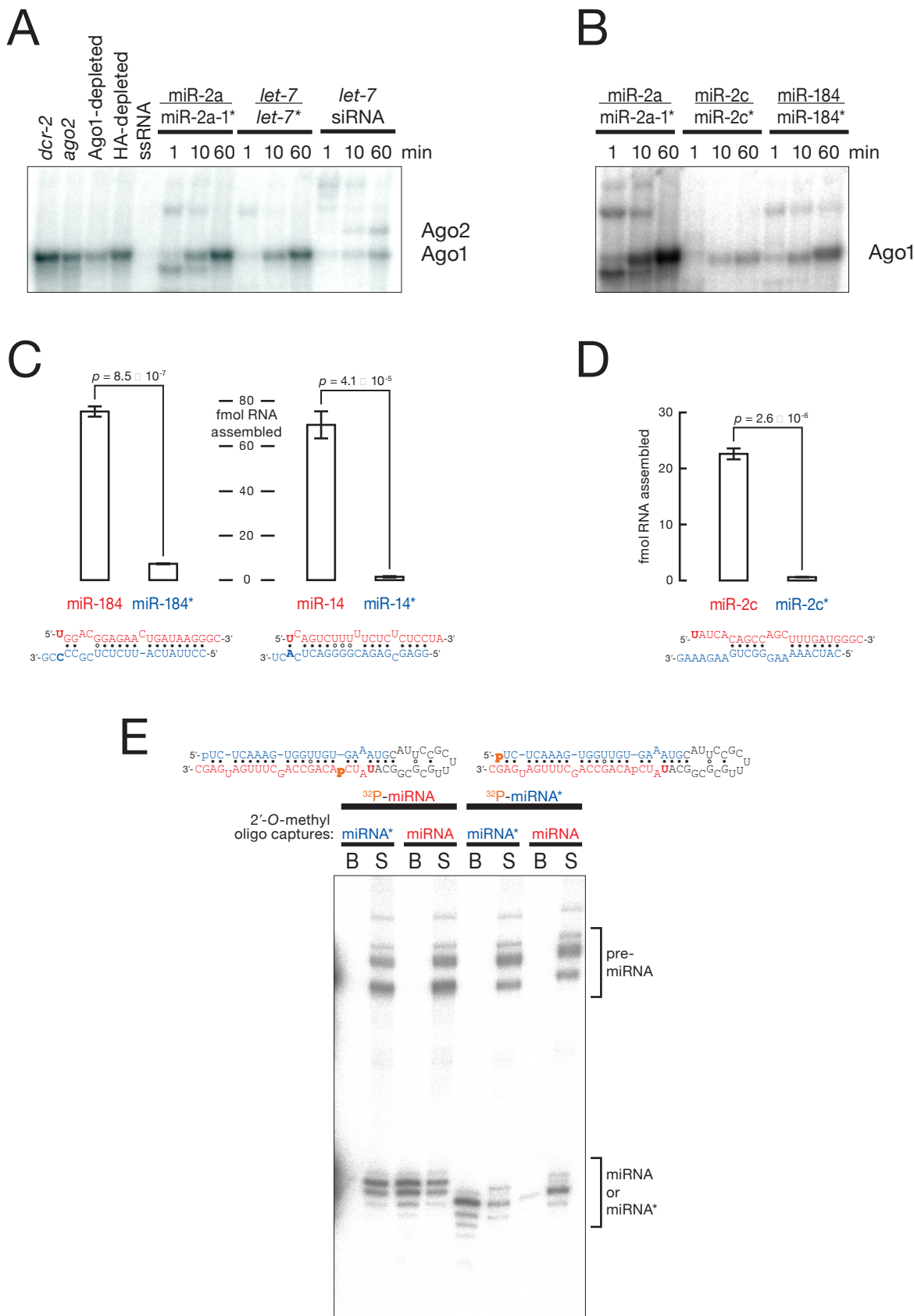

Supplement: Additional file 3 — Figure S3. Faithful in vitro reconstitution of miRNA loading. (A and B) miR-2a, let-7, miR-2c and miR-184 are correctly loaded into Ago1 in fly embryo lysate, and a let-7/anti-let-7 small interfering RNA is correctly loaded into Ago2. Left: lysate prepared from embryos from dcr-2L811fsX- and ago2414-mutant mothers; Ago1-depleted and HA-depleted, wild-type embryo lysate-immunodepleted using anti-Ago1 or anti-HA (hemagglutinin epitope) antibody. Lysate was incubated for one hour with 5′-32P-radiolabeled Drosophila melanogaster miR-2a paired with 5′ phosphorylated miR-2a-1*. Single-stranded (ssRNA), 5′-32P-radiolabeled miR-2a was incubated for one hour in embryo lysate. Each sample was cross-linked using 254 nm ultraviolet light. (C and D) miRNA/miRNA* asymmetry is recapitulated in vitro. (E) In S2 cells, pre-miR-2a-1 liberates both miRNA and miRNA*, and both strands are efficiently loaded into RNA-induced silencing complex as we observed in vitro (Figure 3). [file 1758-907X-2-4-S3.PDF]

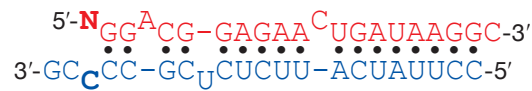

miR-184/miR-184\*

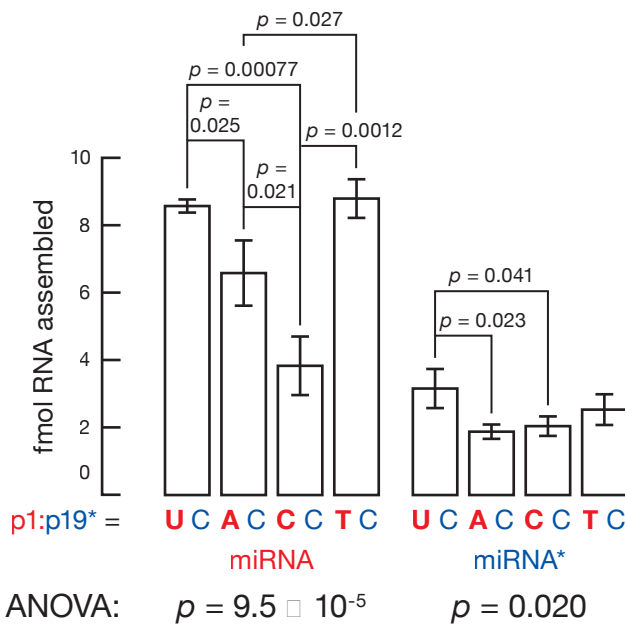

*dcr-2*<sup>L811fsx</sup>

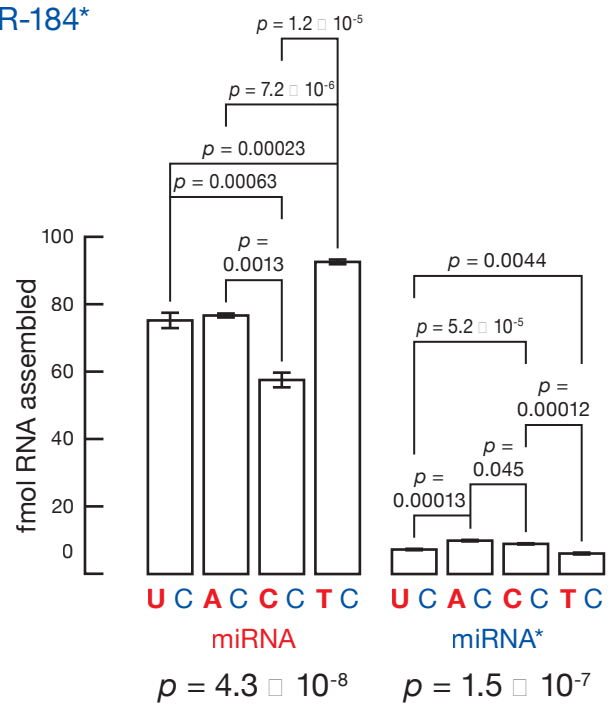

wild-type

Supplement: Additional file 4 — Figure S4. The Ago2 loading machinery has a moderate effect on miR-184 loading preferences, while it strongly affects miR-184* loading preferences. Left: miR-184 and miR-184* capture assay in dcr-2L811fsX-null mutant embryo lysate. Right: miR-184 and miR-184* capture assay in wild-type lysate. [file 1758-907X-2-4-S4.PDF]

A

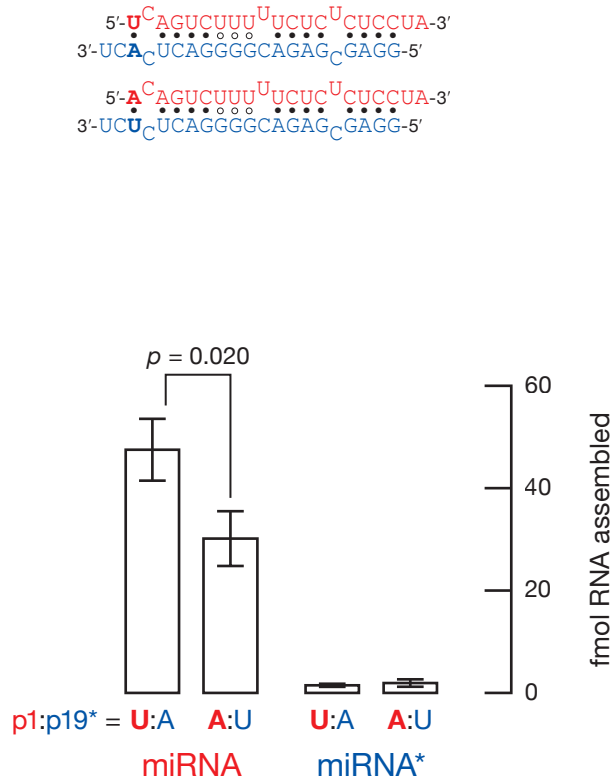

B

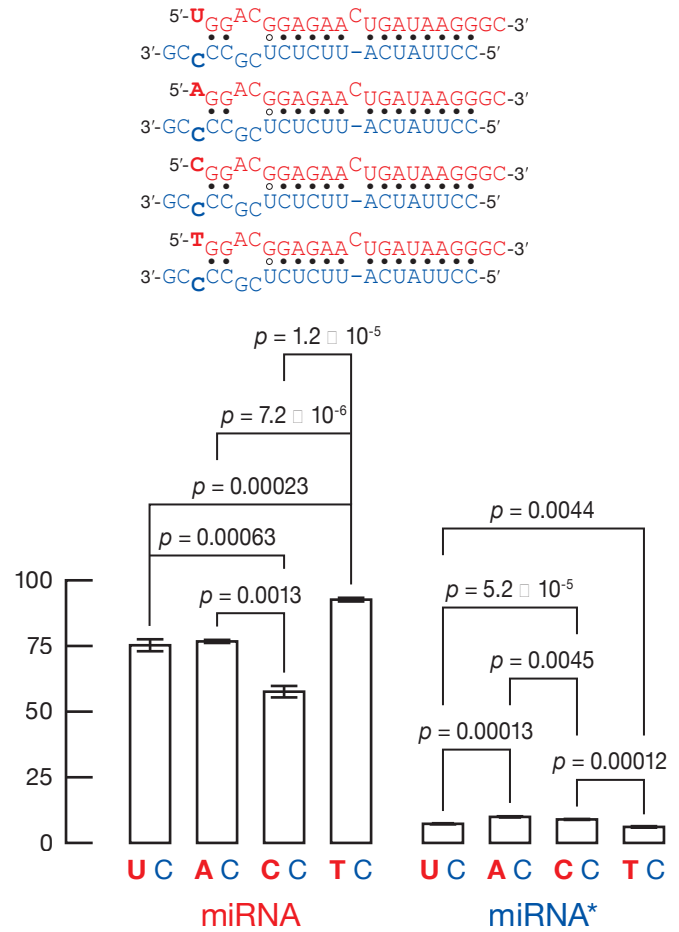

C

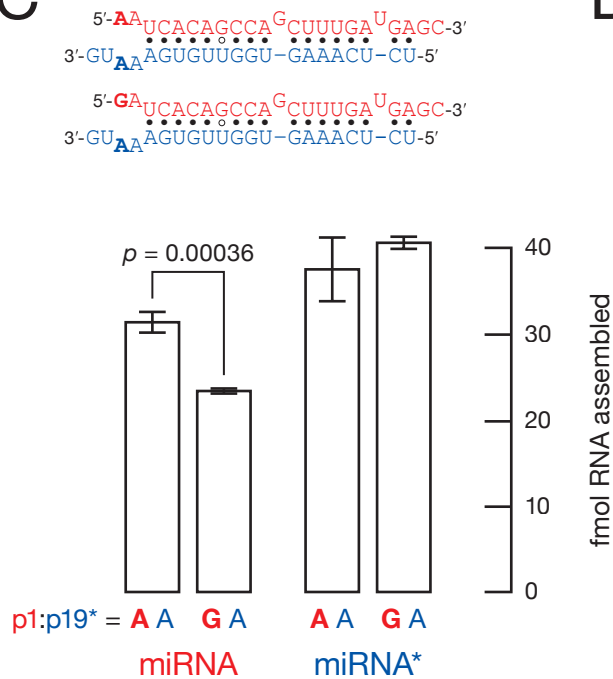

D

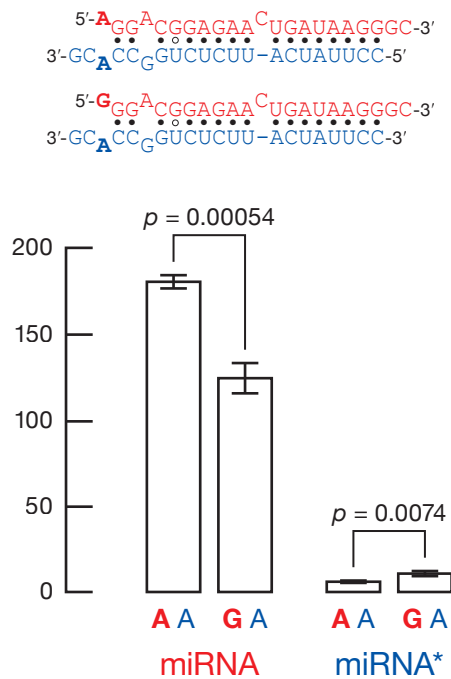

Supplement: Additional file 5 — Figure S5. Identity of miRNA nucleotide 1 affects duplex asymmetry. (A) Changing the 5′-uridine (5′-U) of miR-14 to 5′-adenosine (5′-A) decreased miRNA loading. (B) Changing the 5′-U of miR-184 into 5′-cytidine (5′-C) decreased miRNA loading; mutating it to ribothymidine increased miRNA loading. Changing the 5′-nt of miR-2a (C) or miR-184 (D) into 5′-guanidine (5′-G) decreases miRNA loading (relatively to a 5′-A). [file 1758-907X-2-4-S5.PDF]

**A**

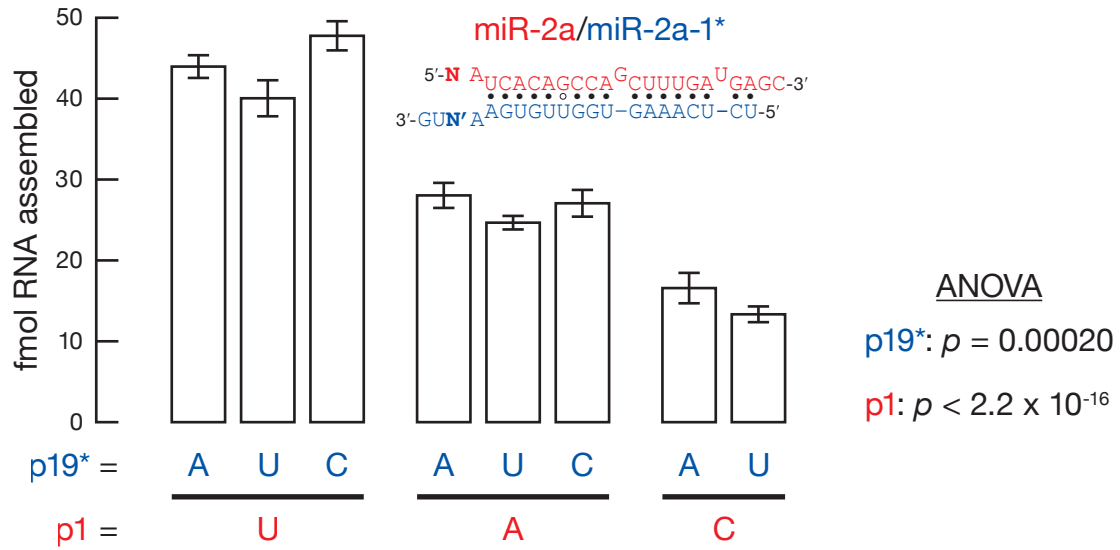

**B**

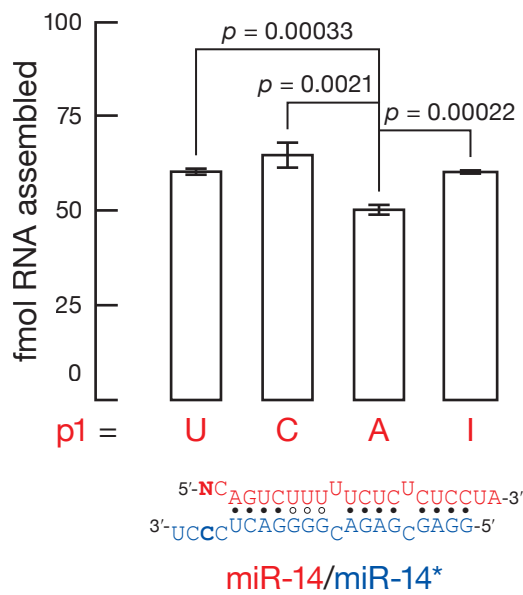

**C**

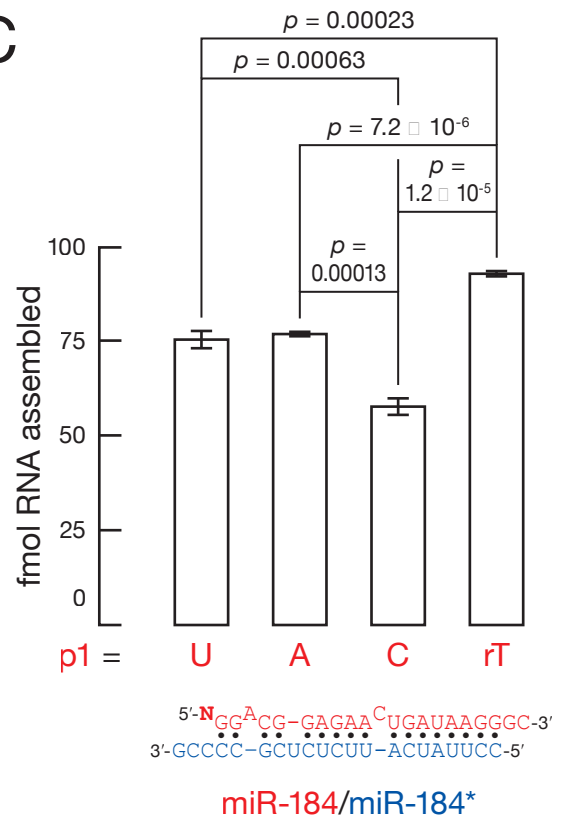

Supplement: Additional file 6 — Figure S6. Duplex-specific order of preference on the identity of the first nucleotide. (A) Regardless of the identity of the facing (p19*) nucleotide, miR-2a is better loaded if it starts with a U than if it starts with an A than if it starts with a C (U > A > C). (B) miR-14 is better loaded if it starts with a U or a C than if it starts with an A (U ~ C > A). (C) miR-184 is better loaded if it starts with a ribothymidine than if it starts with a U or an A than if it starts with a C (T > U ~ A > C). [file 1758-907X-2-4-S6.PDF]

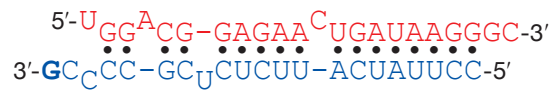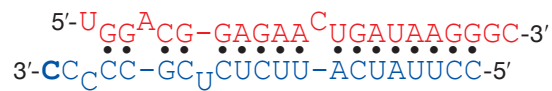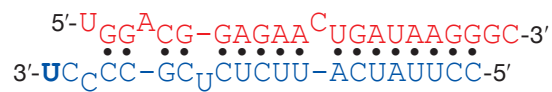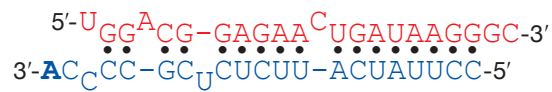

miR-184/miR-184\*

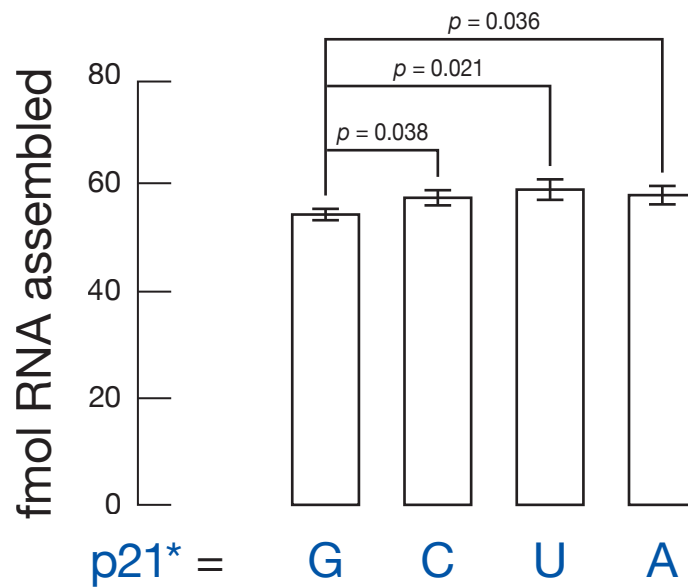

Supplement: Additional file 7 — Figure S7. The sequence of the miRNA* 3′ overhang is not responsible for miRNA-specific preferences for nt 1. The modest effect of the identity of the miRNA* 3′-most nucleotide does not correlate with the base-pairing ability of miRNA nt 1 to the miRNA* 3′ terminus. [file 1758-907X-2-4-S7.PDF]

## Mouse

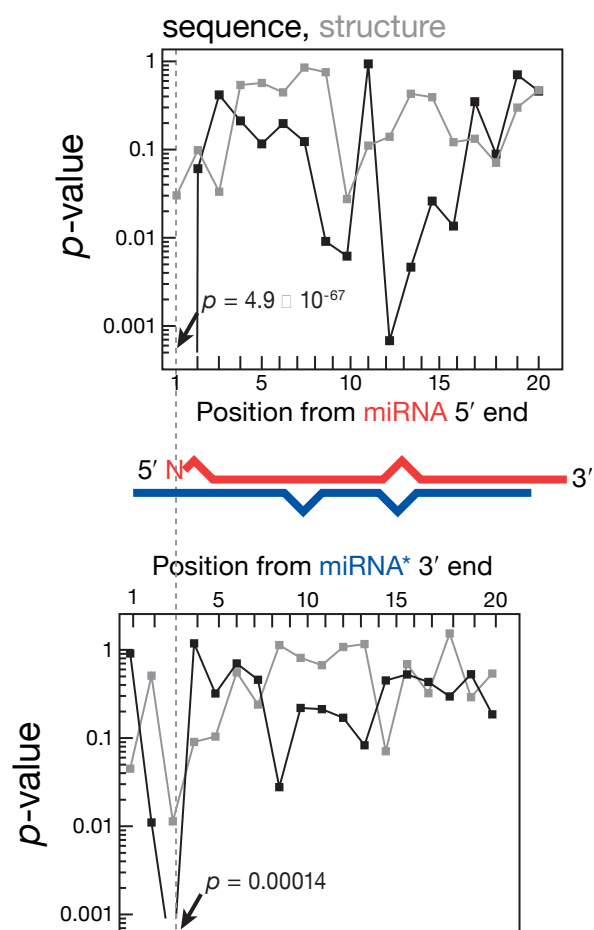

## *C. elegans*

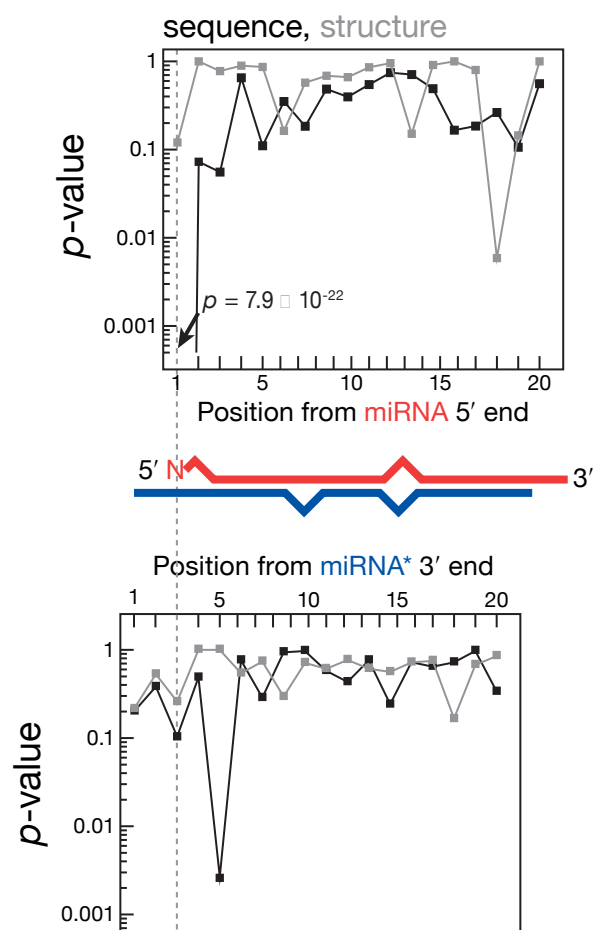

Supplement: Additional file 8 — Figure S8. Structure and sequence features covarying with the identity of miRNA nt 1 in C. elegans and mouse. See Figure 4A legend for details. (A) Covariation in Mus musculus miRNA/miRNA* duplexes. (B) Covariation in C. elegans miRNA/miRNA* duplexes. [file 1758-907X-2-4-S8.PDF]

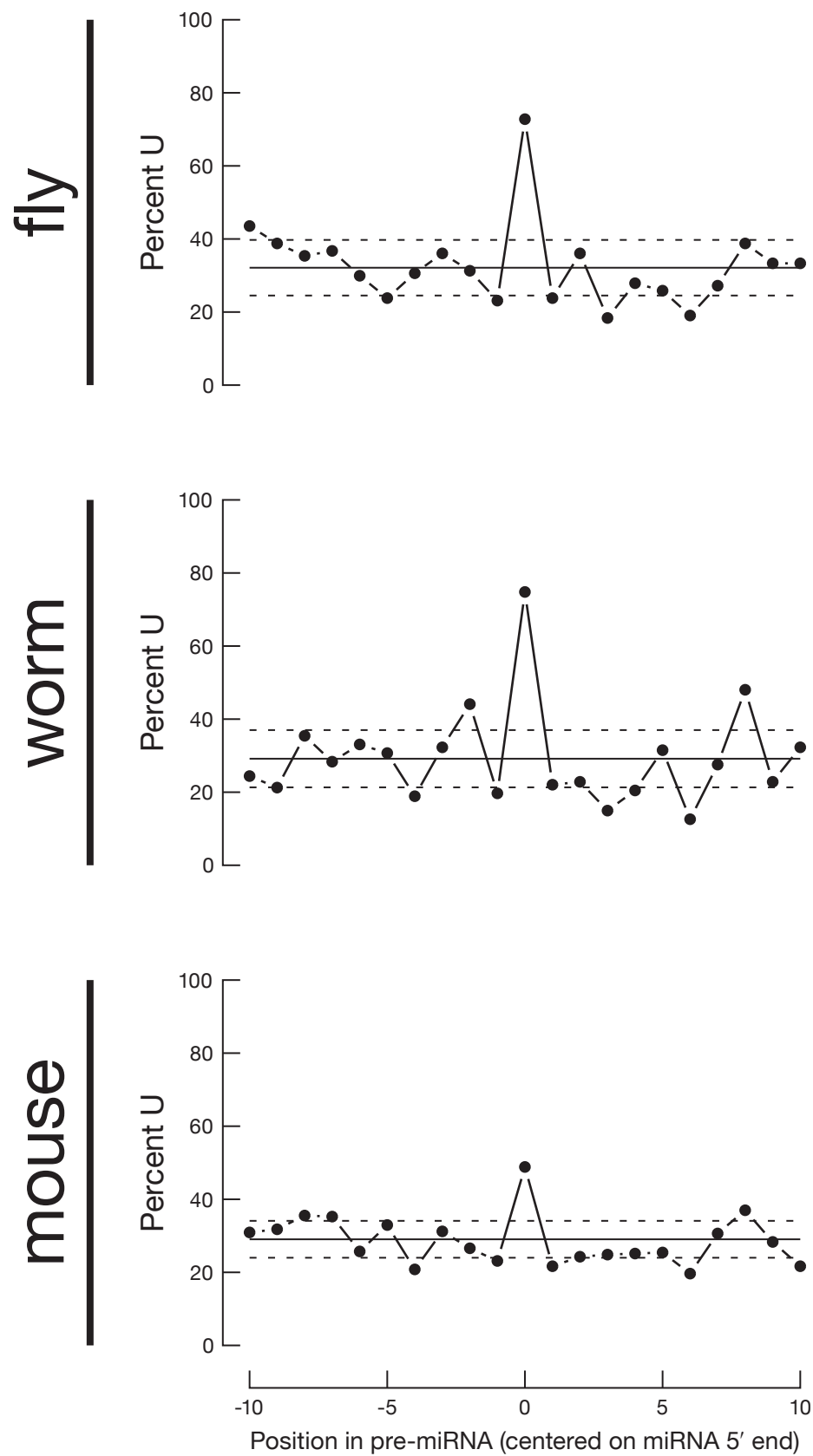

Supplement: Additional file 9 — Figure S9. Pre-miRNA nucleotides flanking miRNA nt 1 are depleted of U. U frequency was measured in pre-miRNA covered by at least 100 reads in the pooled deep-sequencing libraries. The 5′-most nucleotide of mature miRNA is enriched in U (position 0 on the x-axis), while its flanking nucleotides are depleted. The horizontal line indicates the mean U frequency in 100 random sets of nucleotides picked from the corresponding 21-nt segment in the analyzed pre-miRNA. Dashed lines indicate 95% confidence intervals. [file 1758-907X-2-4-S9.PDF]
